# Molecular Diversity of ESBL-Producing *Escherichia coli* from Foods of Animal Origin and Human Patients

**DOI:** 10.3390/ijerph17041312

**Published:** 2020-02-18

**Authors:** Ángel Alegría, Marta Arias-Temprano, Isabel Fernández-Natal, Jose M. Rodríguez-Calleja, María-Luisa García-López, Jesús A. Santos

**Affiliations:** 1 Department of Food Hygiene and Food Technology, Veterinary Faculty, Universidad de León, ES24071 León, Spain; a.alegria@unileon.es (Á.A.); jm.rcalleja@unileon.es (J.M.R.-C.); mlgarl@unileon.es (M.-L.G.-L.); 2 Department of Clinical Microbiology, Complejo Asistencial Universitario de León (CAULE), ES24071 León, Spain; mariast@saludcastillayleon.es (M.A.-T.); ifernandezn@saludcastillayleon.es (I.F.-N.)

**Keywords:** ESBL-producing *E. coli*, β-lactamase genes, clonal diversity, MLST, PFGE

## Abstract

Dissemination of enterobacteria that produce extended spectrum β-lactamases (ESBL) throughout the food chain has become an important health concern. This work aimed to evaluate the occurrence of ESBL-producing bacteria in foods of animal origin and to investigate the similarities between food and human isolates. The presence of beta-lactam-resistant Enterobacteriaceae was analyzed in 108 food samples, isolating 10 strains of *Escherichia coli*, one strain of *Citrobacter freundi*, and one of *Hafnia alvei. E. coli* isolates were compared to a group of 15 strains isolated from human patients by antibiotic susceptibility testing, characterization of ESBL genes (*bla*_TEM_, *bla*_CTX_,), multilocus sequence typing (MLST) and pulse-field gel electrophoresis (PFGE). Nineteen (14 clinical and five food) isolates carried *bla*_CTX_, 14 (six clinical and eight food) carried *bla*_TEM_, and three (one clinical and two food) carried *bla*_SHV_ gen. MLST analysis revealed the prevalence of ST131 among the clinical strains, which grouped together in a PFGE cluster. Food isolates showed higher diversity and two of them (ST57) grouped with clinical strains, whereas another two belonged to clonal groups with virulence potential (ST59). In conclusion, the results showed that foods of animal origin must be regarded as a reservoir of ESBL-producing bacteria of clinical relevance, which might spread through the food chain.

## 1. Introduction

The production of extended spectrum β-lactamases (ESBL) by members of the family Enterobacteriaceae has become an important public health concern. ESBL are plasmid-encoded enzymes that confer resistance to the penicillins; to first-, second-, third-, and fourth-generation cephalosporins; and to aztreonam (but not the cephamycins or carbapenems) [1,2]. Person-to-person transmission of ESBL-producing Enterobacteriaceae has been demonstrated in hospital and community settings, indicating that human colonization is a reservoir for spreading [3]. Furthermore, there are numerous reports on the isolation of ESBL-producing bacteria from foods and food animals, suggesting the possible role of the food production chain as a reservoir for this group of bacteria [1,3,4,5,6,7,8].

The occurrence of ESBL producers is generally high in livestock (up to 60 % of positive chicken fecal samples), whereas low prevalence is found among food samples [9]. In spite of this, some studies found high rates of colonization by ESBL-producers in chicken meat [10,11] and contamination of raw cow’s milk has been also reported [6,12]. There are reports of a strong correlation between the presence of ESBL-producing bacteria in foods and the incidence of infections in humans, and it may be assumed that food of animal origin may be contaminated with resistant bacteria, thus contributing to spreading within the human population [1,4]. Moreover, the resistant bacteria can carry other virulence-related genes; it is noteworthy that strains of Shiga toxin-producing *Escherichia coli* (STEC), which are related to ruminants and considered foodborne pathogens, have been demonstrated as ESBL-producers, indicating that the transfer of the ESBL plasmid from commensals to foodborne pathogenic strains is possible [1,13,14].

Available studies show that ESBL-producing food isolates present a low level of similarity compared with human isolates, but some clonal overlaps are sometimes revealed, thus more research is required to understand the molecular relatedness, the possible reservoirs, and the role of the food chain in the transmission of ESBL-producing bacteria [15,16,17,18,19].

The aim of this work is to evaluate the occurrence of ESBL-producing bacteria in foods of animal origin (milk and dairy products and chicken meat) and to investigate the similarities between food and human isolates. This information could be of help in monitoring and controlling the dissemination of this concerning group of bacteria.

## 2. Materials and Methods

### 2.1. Samples and Microorganisms

A total of 108 food samples of animal origin (68 samples of goat’s milk, 10 samples of ewe’s milk, 20 samples of fresh cheese, and 10 samples of chicken meat) were collected. In total, 10 g or 10 mL of each sample was inoculated into 90 mL of Tryticase soy broth plus 0.6 % yeast extract (TSBYE) and cultured at 42 °C for 18 h. An aliquot of this enrichment culture was streaked onto Chromagar ESBL (Chromagar, Paris, France) and incubated at 37 °C for 24 h. Dark pink to reddish and metallic blue colonies were picked and cultured to purify. Fifteen clinical isolates of ESBL-producing E. coli were kindly provided by the Department of Clinical Microbiology of the Complejo Asistencial Universitario de León (CAULE). All the isolates were preserved at −40 °C in Trypticase soy broth (TSB) plus 40% glycerol for further characterization.

### 2.2. Matrix-Assisted Laser Desorption Ionization Time-of-Flight (MALDI-TOF) Identification of Isolates

The isolates were grown on TSA for 16–24 h at 37 °C. Colony material was collected with a sterile pipette tip and smeared as a thin film on a MALDI target plate. After air drying, each sample was overlaid with 0.5 µL of the matrix solution (α-Cyano-4-hydroxycinnamic acid, CHCA) and allowed to dry. Spectra were acquired with the MALDI Biotyper system (Bruker Daltonik, Bremer, Germany) and compared with the reference database (Bruker Daltonik).

### 2.3. Antimicrobial Resistance Characterization

Phenotypic confirmation of ESBL production was carried out by combination disk test using both cefotaxime and ceftazidime disks, alone and in combination with clavulanic acid (Condalab). Minimum inhibitory concentrations were obtained with the MicroScan WalkAway system (Siemens Healthcare Diagnostics Inc., West Sacramento, CA, USA) using the NEG-MIC Type 44 panel. PCR detection of blaTEM, blaSHV, and blaCTX-M genes was done using the primers and conditions described by Monstein et al. [20]. Subtyping of CTX-M group was carried out by sequencing and comparison with AMRFinderPlus tool available from the National Center for Biotechnology Information (NCBI) server [21].

### 2.4. Phylogenetic Group and Multilocus Sequence Typing (MLST) Analysis

Phylogenetic groups were determined by the quadruplex PCR method [22]. Multilocus sequence typing was carried out by amplifying and sequencing seven conserved housekeeping genes (adk, fumC, gyrB, icd, mdh, purA, and recA), as recommended by the EnteroBase Database (http://mlst.warwick.ac.uk/mlst/dbs/Ecoli). PCR products were sequenced in a MegaBACE 500 sequencer (Amersham Biosciences, Piscataway, NJ, USA). Raw sequences were visually reviewed and edited using the Chromas Lite 2.1 software (Technelysium, South Brisbane, Australia) and aligned with the ClustalW algorithm of the MEGA7 software [23]. Each gene locus was assigned an allele number and a sequence type (ST) was determined for each isolate according to the allele profile. Grouping of isolates into clonal complexes was done with the eBurst algorithm implemented in the EnteroBase platform.

### 2.5. Pulse Field Gel Electrophoresis (PFGE)

Genomic DNA for PFGE was prepared following the protocol proposed by PulseNet (https://www.cdc.gov/pulsenet/index.html). DNA digestion with XbaI and PFGE were carried out as already described [24]. Comparison of PFGE profiles was done with the GelCompar 6.6 software (St. Martens Latem, Belgium). Similarities were obtained using the Dice coefficient at 0.5% optimization and 1% tolerance and a dendrogram was constructed with the Unweighted Pair Group Method with Arithmetic mean (UPGMA) clustering method.

## 3. Results

Of the 108 samples analyzed, 13 (12.0%) showed positive growth of colored colonies on Chromagar ESBL, including five from ewe’s milk, three from goat‘s milk, three from fresh cheese, and two from chicken meat. MALDI-TOF analysis identified 10 isolates (76.9%) as *E. coli*, obtained from ewe and goat’s milk and chicken meat; and identified two (15.4%) as *Citrobacter freundi* and one (7.7%) as *Hafnia alvei*, which were obtained from fresh cheese. In total, *E. coli* isolates were recovered from 10 out of 108 food samples (9.3%), *Citrobacter freundii* was recovered from 2 (1.8%), and *Hafnia alvei* from one (0.9%). All the clinical isolates showed a dark pink color on Chromagar ESBL and were identified as *E. coli*.

All the *E. coli* colonies were resistant to cefotaxime or ceftazidime and showed larger inhibition zones if clavulanic acid was present; however, three food isolates obtained from ewe’s milk turned out to not be ESBL-producers, as the inhibition zones in the presence of clavulanic acid showed less than five mm increase. These results were confirmed by the Microscan system, where the isolates showed less than three twofold concentration decrease in the minimal inhibitory concentration (MIC) for cefotaxime in combination with clavulanate compared with the MIC for cefotaxime alone (Table 1). Both clinical and food isolates were sensitive to carbapenems and aminoglycosides. Additionally, all the isolates were sensitive to colistin. In general, clinical isolates were resistant to beta-lactams, cephalosporins, and fluoroquinolones, whereas food isolates were less resistant to fluoroquinoles and two isolates obtained from ewe’s milk were sensitive to the majority of third-generation cephalosporins (Table 1 and Table S1). Percentages of resistance against different antimicrobials from each isolate collection are shown in Table 2.

Nineteen (14 clinical and five food) isolates carried *bla*_CTX-M_ genes, 14 (six clinical and eight food) isolates carried *bla*_TEM_ genes, and three (one clinical and two food) isolates carried *bla*_SHV_ genes. Eight (five clinical and three food) isolates carried both TEM and CTX-M genes and three (one clinical and two food) isolates carried *bla*_TEM_ and *bla*_SHV_ genes. Genes belonging to the CTX-M-1 group were detected in 12 clinical isolates, the blaCTX-M-15 gene was detected in ten isolates, and blaCTX-M-1 gene was detected in two isolates. The remaining seven CTX-M-positive strains carried genes from the CTX-M-9 group, with the bla-CTX-M-14 gene in two clinical and three food isolates and the blaCTX-M-9 gene in two food isolates (Table 1). The three *bla_SHV_* genes belonged to the SHV-12 group, which has been detected in chicken samples in Spain [25].

Twenty-two isolates were grouped in eight clonal complexes by MLST analysis. Eleven clinical strains were included in CC131 and the remaining four clinical strains were included in CC23, CC86, CC648, and one was not assigned to any CC. The food isolates were included in CC59 (two isolates), CC155 (one isolate), CC350 (4 isolates), and CC1689 (one isolate), whereas two isolates were not assigned to any CC. Among clinical isolates, eight different STs were detected, with ST131 being the most frequent (eight isolates). The remaining seven isolates were assigned to four unique STs, ST88, ST117, ST3136, and ST7519; and three new STs, related to ST86, ST131, and ST648, respectively. The food isolates were assigned to nine STs, including ST57 (four isolates), ST59, ST155, ST345, ST373, ST447, and a new ST related to ST59, with one isolate each (Table 3).

Eight clinical isolates belonged to phylogenetic group B2, all of which were included in CC131, four were included in phylogroup D, two in phylogroup A, and one in B1. Phylogroup B2 was not detected among food isolates, which were assigned to groups D (six isolates), B1 (three isolates), and A (one isolate) (Table 3).

**Figure 1:**
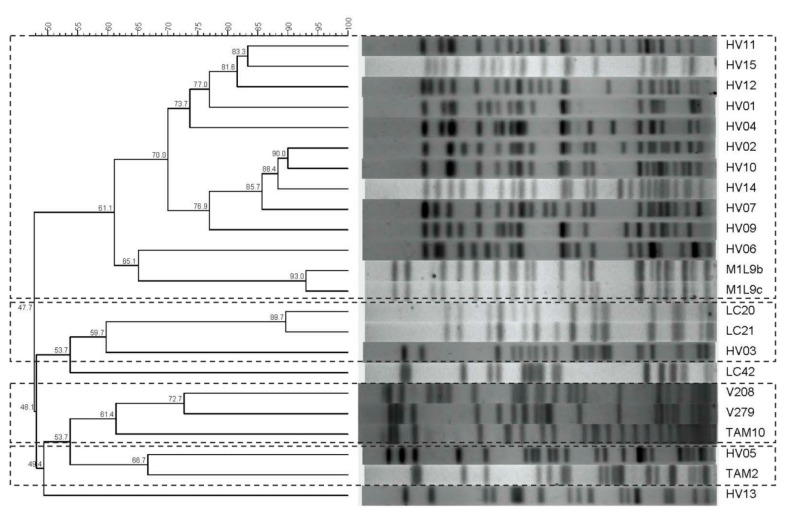
PFGE profiles and cluster analysis of 23 human and food isolates. Main groups are marked by broken lines.

PFGE typing of isolates resulted in 23 patterns, which grouped into four clusters with a minimum cutoff value of 59.7 %. Cluster 1 included 11 clinical isolates, 10 of which were from CC131 and one from CC86, as well as two food isolates of CC350. Cluster 2 was formed by one clinical isolate and two food isolates, both obtained from goat’s milk. All the isolates grouped in clusters 1 and 2 carried the CTX gene. Cluster 3 included three food isolates and cluster 4 included one clinical isolate and one food isolate. One clinical isolate and one food isolate were not included in any cluster and one clinical isolate and one food isolate were non-typeable by this method (Figure 1 and Table 3).

## 4. Discussion

Foods of animal origin are increasingly reported as carriers of β-lactam-resistant enterobacteria. In this study, the presence of ESBL-producers was in agreement with other reports, which showed similar percentages of prevalence [6,16]. The identification of species other than *E. coli* has been also reported in milk and dairy products and in chicken meat [6,11,16]. Detection of suspected ESBL-producers, which turned out to be non-ESBL, has been noticed for milk isolates as well [6,26], but it must be taken into account that the isolates found in this work are resistant to third generation cephalosporins and that the inhibition zones are augmented in the presence of clavulanic acid; moreover, the isolates carried ESBL-related genes, thus they can act as a reservoir of antibiotic resistance genes.

As expected, the majority of the clinical isolates were producers of CTX-M 15 β-lactamases, as well as CTX-M 14 (Table 1). The spreading of the CTX-M 14 gene, belonging to the CTX-M group 9, in clinical samples in Spain is well-known [27,28], and is also frequently found in broilers and poultry meat [10,16,29]; however, the presence of CTX-M group 9 genes in milk and milk animals is scarcely reported and CTX-M group 1 genes are commonly found in those samples [6,12,26,30]. The three isolates carrying TEM genes alone did not express the ESBL phenotype, suggesting the presence of a broad spectrum of non-ESBL β-lactamase, probably TEM-1, which is commonly found in Enterobacteriaceae and has been detected in food-producing animals in other studies [31,32].

MLST analysis revealed the prevalence of CC131 among the clinical strains. The majority of CC131 strains were assigned to phylogenetic group B2 and produced CTX-M 15 β-lactamase, indicating that they belong to the worldwide predominant pathogenic clone [33], with only two isolates assigned to group D and one isolate to group A. Moreover, all the ST131 isolates grouped together in a PFGE cluster (Figure 1). One clinical isolate was assigned to CC648, which is spread among different niches in Europe [16]. Food isolates showed higher diversity than clinical ones, with the detection of four isolates of CC350 (ST57) assigned to phylogenetic group D being remarkable; they were isolated from ewe’s milk, and two of them (producers of CTX-M 9) grouped together with clinical strains in a PFGE cluster, whereas the other two isolates carried the TEM gene and were not considered ESBL producers (Figure 1). Two isolates from goat’s milk belonged to a clonal group related to D-ST59/ST59 and were producers of CTX-M 14, which is an emerging clonal group with high virulence potential [34].

## 5. Conclusions

In conclusion, the results of the study show that the presence of ESBL producers in milk, dairy products, and chicken meat is not rare, and some of them belong to emerging clones of clinical relevance. The genetic profiles of food isolates were different from clinical isolates; however, goat and ewe’s milk and chicken meat must be regarded as a reservoir of ESBL-producing bacteria that might spread through the food chain.

## Figures and Tables

**Table 1:** Genetic characteristics and antimicrobial susceptibilities of extended spectrum β-lactamases (ESBL)-producing *E. coli* from human and food origins.

		ESBL Genes				Antimicrobial Susceptibilities (µg/mL) ^1,2^																		
Strain	Source	TEM	SHV	CTX	CTX Group	AMP	AMC	TZP	CEF	CTX	C/C	FOX	AZT	CIP	GM	TO	AK	TIG	TET	ETP	IMP	NAL	FOF	SXT
HV1	Human			15	1	>16	S	S	>16	>32	S	S	>16	>2	S	S	S	S	>8	S	S	>16	S	>4/76
HV2	Human			15	1	>16	16/8	S	>16	>32	S	S	>16	>2	>8	>8	S	S	S	S	S	>16	S	S
HV3	Human	+		1	1	>16	>16/8	16	>16	>32	S	S	16	>2	>8	>8	S	S	>8	S	S	>16	S	>4/76
HV4	Human			14	9	>16	S	S	>16	>32	S	S	>16	>2	S	S	S	S	S	S	S	>16	S	S
HV5	Human	+		15	1	>16	S	S	>16	>32	S	S	4	S	S	S	S	S	>8	S	S	S	S	S
HV6	Human	+		15	1	>16	16/8	S	>16	>32	S	S	>16	>2	S	>8	S	S	S	S	S	>16	>64	>4/76
HV7	Human			15	1	>16	16/8	S	>16	>32	S	S	>16	>2	S	>8	S	S	S	S	S	>16	S	>4/76
HV8	Human	+	+			>16	S	S	>16	16	S	S	>16	>2	S	S	S	S	>8	S	S	>16	S	>4/76
HV9	Human			15	1	>16	>16/8	16	>16	>32	S	S	>16	>2	S	>8	16	S	>8	S	S	>16	S	4/76
HV10	Human			15	1	>16	16/8	S	>16	>32	S	S	>16	>2	>8	>8	S	S	>8	S	S	>16	S	>4/76
HV11	Human	+		15	1	>16	S	S	>16	>32	S	S	>16	>2	S	S	S	S	S	S	S	>16	S	S
HV12	Human	+		14	9	>16	16/8	S	>16	>32	S	S	16	>2	>8	>8	S	S	>8	S	S	>16	S	≥2/38
HV13	Human			1	1	>16	8/4	S	>16	>32	S	S	>16	>2	S	S	S	S	>8	S	S	>16	S	S
HV14	Human			15	1	>16	>16/8	>64	>16	>32	S	S	>16	>2	>8	S	S	S	S	S	S	>16	64	S
HV15	Human			15	1	>16	16/8	S	>16	>32	S	S	>16	>2	S	>8	S	S	S	S	S	>16	S	>4/76
LC20	Goat’s milk			14	9	>16	S	S	>16	>32	S	S	8	S	S	S	S	S	S	S	S	S	S	S
LC21	Goat’s milk			14	9	>16	S	S	>16	>32	S	S	4	S	S	S	S	S	S	S	S	S	S	S
LC42	Goat’s milk	+		14	9	>16	S	S	>16	>32	S	S	16	S	S	S	S	S	>8	S	S	S	S	S
M1L9B	Ewe’s milk	+		9	9	>16	S	S	>16	>32	S	16	8	>2	4	S	S	S	>8	S	S	>16	S	>4/76
M1L9C	Ewe’s milk	+		9	9	>16	S	S	>16	>32	S	16	4	>2	S	S	S	S	>8	S	S	>16	S	>4/76
V208	Ewe’s milk	+				S	S	S	S	S	S	S	S	S	S	S	S	S	>8	S	S	S	S	S
V279	Ewe’s milk	+				>16	S	S	16	S	S	S	S	S	S	S	S	S	S	S	S	S	S	S
V298	Ewe’s milk	+				S	S	S	S	S	S	S	S	S	S	S	S	S	S	S	S	S	S	S
TAM2	Chicken meat	+	+			>16	S	S	>16	8	S	S	>16	S	S	S	S	S	>8	S	S	S	S	S
TAM10	Chicken meat	+	+			>16	16/8	S	>16	>32	S	16	>16	>2	S	S	S	S	>8	S	S	>16	S	S

^1^ AMP, Ampicillin; AMC, Amoxicillin clavulanic acid; TZP, Piperacillin tazobactam; CEF, Cefalotina; CTX, Cefotaxime; C/C, Cefotaxime clavulanic acid; FOX, Cefoxitin; AZT, Aztreonam; CIP, Ciprofloxacin; GM, Genatmicin; TO, Tobramycin; AK, Amikacin; TIG, Tygecycline; TET, Tetracycline; ETP, Ertapenem; IMP, Imipenem; NAL, Nalidixic acid; FOF, Fosfomycin; SXT, Trimethoprim-sulfamethoxazole. ^2^ S = Susceptible. Susceptibility limits (µg/mL): AMP, ≤8; AM.C, ≤8; TZP, ≤8; CEF, ≤8; CTX, ≤1; C/C, ≤0.5; FOX, ≤8; AZT, ≤1; CIP, ≤0.5; GM, ≤2, TO, ≤2; AK, ≤8, TIG, ≤1; TET, S; ETP, ≤0.5, IMP, ≤1; NAL, ≤16, FOF, ≤32; SXT, ≤2/38.

**Table 2:** Percentages of resistance against different antimicrobials from each isolate collection.

	Antibiotic ^1^																		
	AMP	AMC	TZP	CEF	CTX	C/C	FOX	AZT	CIP	GM	TO	AK	TIG	TET	ETP	IMP	NAL	FOF	SXT
Human	100	60	20	100	100	0	0	100	93.3	33.3	53.3	6,6	0	53.3	0	0	93.3	13.3	60
Food	80	10	0	80	70	0	30	70	30	10	0	0	0	60	0	0	30	0	20

^1^ AMP, Ampicillin; AMC, Amoxicillin clavulanic acid; TZP, Piperacillin tazobactam; CEF, Cefalotina; CTX, Cefotaxime; C/C, Cefotaxime clavulanic acid; FOX, Cefoxitin; AZT, Aztreonam; CIP, Ciprofloxacin; GM, Genatmicin; TO, Tobramycin; AK, Amikacin; TIG, Tygecycline; TET, Tetracycline; ETP, Ertapenem; IMP, Imipenem; NAL, Nalidixic acid; FOF, Fosfomycin; SXT, Trimethoprim-sulfamethoxazole.

**Table 3:** Genetic characterization of human and food *E. coli* isolates. PFGE, pulse field gel electrophoresis.

Strain	Source	Phylogenetic Group	Sequence Type	Clonal Complex	PFGE
HV1	Human	B2	ST131	131	1
HV2	Human	B2	ST131	131	1
HV3	Human	A	ST88	23	2
HV4	Human	B1	ST86 related	86	1
HV5	Human	B2	ST3136	131	4
HV6	Human	B2	ST7519	131	1
HV7	Human	B2	ST131	131	1
HV8	Human	D	ST648 related	648	NT
HV9	Human	B2	ST131	131	1
HV10	Human	B2	ST131 related	131	1
HV11	Human	D	ST131	131	1
HV12	Human	D	ST131	131	1
HV13	Human	D	ST117		
HV14	Human	B2	ST131	131	1
HV15	Human	A	ST131	131	1
LC20	Goat’s milk	D	ST59 related	59	2
LC21	Goat’s milk	D	ST59	59	2
LC42	Goat’s milk	B1	ST155	155	
M1L9B	Ewe’s milk	D	ST57	350	1
M1L9C	Ewe’s milk	D	ST57	350	1
V208	Ewe’s milk	D	ST57	350	3
V279	Ewe’s milk	D	ST57	350	3
V298	Ewe’s milk	B1	ST447		NT
TAM2	Chicken meat	A	ST373	1689	4
TAM10	Chicken meat	B1	ST345		3

## Supplementary Materials

The following are available online at https://www.mdpi.com/1660-4601/17/4/1312/s1, Table S1: Genetic characteristics and antimicrobial susceptibilities of ESBL-producing *E.coli* from human and food origins.
