# Supplementary material for: Molecular Diversity of ESBL-Producing Escherichia coli from Foods of Animal Origin and Human Patients
Source: Int J Environ Res Public Health. 2020 Feb 18;17(4):1312. doi: 10.3390/ijerph17041312 (PMC7068493; doi:10.3390/ijerph17041312)
Supplement: Supplementary file 1 [file ijerph-17-01312-s001.pdf]

Supplementary Table S1. Genetic characteristics and antimicrobial susceptibilities of ESBL-producing *E.coli* from human and food origin

		ESBL genes				Antimicrobial susceptibilities (µg/mL)[1,2]													
**Strain**	**Source**	**TEM**	**SHV**	**CTX**	**CTX group**	**SAM**	**FEP**	**CAZ**	**CXM**	**LVX**	**NOR**	**ATM**	**MEM**	**CHL**	**MIN**	**NIT**	**CST**	**DOR**	**PIP**
HV1	Human			15	1	16/8	>16	>16	>16	>4	>1	>16	S	S	S	S	S	S	>64
HV2	Human			15	1	16/8	>16	16	>16	>4	>1	>16	S	S	S	S	S	S	>64
HV3	Human	+		1	1	>16/8	16	4	>16	>4	>1	16	S	>16	>8	S	S	S	>64
HV4	Human			14	9	16/8	>16	>16	>16	>4	>1	>16	S	S	S	S	S	S	>64
HV5	Human	+		15	1	>16/8	>16	S	>16	S	S	4	S	S	S	>64	S	S	>64
HV6	Human	+		15	1	>16/8	16	16	>16	>4	>1	>16	S	S	S	S	S	S	>64
HV7	Human			15	1	>16/8	>16	>16	>16	>4	>1	>16	S	S	S	S	S	S	>64
HV8	Human	+	+			16/8	4	>16	>16	>4	>1	>16	S	>16	S	S	S	S	>64
HV9	Human			15	1	>16/8	>16	>16	>16	>4	>1	>16	S	S	S	S	S	S	>64
HV10	Human			15	1	16/8	>16	>16	>16	>4	>1	>16	S	S	S	S	S	S	>64
HV11	Human	+		15	1	>16/8	>16	>16	>16	>4	>1	>16	S	S	S	S	S	S	>64
HV12	Human	+		14	9	>16/8	>16	2	>16	>4	>1	>16	S	S	S	S	S	S	>64
HV13	Human			1	1	>16/8	>16	16	>16	>4	>1	>16	S	16	>8	S	S	S	>64
HV14	Human			15	1	>16/8	>16	16	>16	>4	>1	>16	S	S	S	S	S	S	>64
HV15	Human			15	1	>16/8	>16	>16	>16	>4	>1	>16	S	S	S	S	S	S	>64
LC20	Goat's milk			14	9	>16/8	>16	S	>16	S	S	8	S	S	S	S	S	S	>64
LC21	Goat's milk			14	9	>16/8	16	S	>16	S	S	4	S	S	S	S	S	S	>64
LC42	Goat's milk	+		14	9	16/8	>16	2	>16	S	S	16	S	S	>8	S	S	S	>64
M1L9B	Ewe's milk	+		9	9	>16/8	>16	S	>16	>4	>1	8	S	16	>8	S	S	S	>64
M1L9C	Ewe's milk	+		9	9	>16/8	>16	S	>16	>4	>1	4	S	16	>8	S	S	S	>64
V208	Ewe's milk	+				S	S	S	8	S	S	S	S	S	S	S	S	S	S
V279	Ewe's milk	+				>16/8	S	S	S	S	S	S	S	S	S	S	S	S	>64

V298	Ewe’s milk	+		S	S	S	8	S	S	S	S	>16	S	S	S	S	S
TAM2	Chicken meat	+	+	16/8	S	>16	16	S	S	>16	S	>16	S	S	S	S	>64
TAM10	Chicken meat	+	+	>16/8	8	>16	>16	>4	>1	>16	S	>16	>8	>64	S	S	>64

[1] SAM, Ampicillin-sulbactam; FEP, Cefepime; CAZ, ceftazidime; CXM, Cefuroxime (axetil or sodium); LVX, Levofloxacin; NOR, Norfloxacin; ATM, Aztreonam; MEM, Meropenem; CHL, Chloramphenicol; MIN, Minocycline; NIT, Nitrofurantoin; TIG, Tygecycline; CST, Colistin; DOR, Doripenem; PIP, Piperacillin.

[2] S = Susceptible. Susceptibility limits (μg/mL): SAM, <=8/4; FEP, <=1; CAZ, <=1; CXM, <=4; LVX, <=1; NOR, <=0,5; ATM <=1; MEM, <=1; CHL, <=8; MIN, <=4; NIT, <=32; CST, S; DOR, <=1; PIP, <=8.
